# Implementation and assessment of the black body bias correction in quantitative neutron imaging

**DOI:** 10.1371/journal.pone.0210300

**Published:** 2019-01-04

**Authors:** Chiara Carminati, Pierre Boillat, Florian Schmid, Peter Vontobel, Jan Hovind, Manuel Morgano, Marc Raventos, Muriel Siegwart, David Mannes, Christian Gruenzweig, Pavel Trtik, Eberhard Lehmann, Markus Strobl, Anders Kaestner

**Affiliations:** 1 Laboratory for Neutron Scattering and Imaging, Paul Scherrer Institut, Villigen, Switzerland; 2 Electrochemistry Laboratory, Paul Scherrer Institut, Villigen, Switzerland; Dartmouth College Geisel School of Medicine, UNITED STATES

## Abstract

We describe in this paper the experimental procedure, the data treatment and the quantification of the black body correction: an experimental approach to compensate for scattering and systematic biases in quantitative neutron imaging based on experimental data. The correction algorithm is based on two steps; estimation of the scattering component and correction using an enhanced normalization formula. The method incorporates correction terms into the image normalization procedure, which usually only includes open beam and dark current images (open beam correction). Our aim is to show its efficiency and reproducibility: we detail the data treatment procedures and quantitatively investigate the effect of the correction. Its implementation is included within the open source CT reconstruction software MuhRec. The performance of the proposed algorithm is demonstrated using simulated and experimental CT datasets acquired at the ICON and NEUTRA beamlines at the Paul Scherrer Institut.

## 1. Introduction

Neutron imaging is a well-established technique for non-destructive investigations in a wide range of scientific areas [1] [2] [3], such as porous media and geology [4] [5] [6], electrochemistry [7] [8] [9], cultural heritage [10] [11][12] and industrial applications [13] [14].

The development of neutron imaging from a non-destructive testing tool [15] towards a key technique in materials science [16] was driven by ever improving resolution and quantification capabilities of the technique [17] [18], in turn further driving the need of quantitative analysis.

To allow for such quantitative analysis, a major role is played by the correction of biases, caused by deviations from the conventional description and measurement of the linear attenuation through the Beer Lambert law:

$$I(x,y,E)=I_{0}(x,y,E)exp(-\int \mu (x,y,E)ds),$$

where *I* and *I*_*0*_ are the attenuated and incident intensities, μ is the linear attenuation coefficient of the materials along the straight path, *s*, through the sample and E refers to the energies of the neutron spectrum.

Main contributions for deviations from the theoretical behavior are not accounted for straightforwardly in conventional measurements and analyses, therefore causing biases in quantification. One of those can be identified as the spectral influence through distinct scattering behavior [19][20] leading to effects comparable to beam hardening in x-ray imaging [21]. An appropriate solution to this is for example narrowing down the beam spectrum to eventually monochromatic neutron imaging [22] [23], which however comes at the expense of significantly decreased flux and increased exposure times, or alternatively applying dedicated correction algorithm at the post processing stage [24].

Another key bias, which we will deal with here, is introduced by scattered neutrons from interaction with the sample and the experimental set-up, as well as of secondary radiation in the detection process. The multitude of effects and their nature hinder a proper analytical correction. To deal with these effects, we have recently proposed [25] an experimental method for improving the quantification of neutron imaging measurements by reducing the neutron scattering and other systematic biases in scintillator-camera based detectors. The main idea is to acquire reference measurements using a grid of neutron absorbers (called black bodies, BB) to measure directly the additive biases and sample scattering, with a minimal increase of experiment time (4%). The method proved to efficiently remove background in two test samples with moderate (20%) and very low (1%) transmission.

In previous research, the probability density of scattered neutrons is mainly estimated through Monte Carlo simulation software [26] [27] [19] [28] [29] thus requiring a priori knowledge of the neutron energy spectrum, the detector properties, the experimental setup and the sample characteristics (material and thickness). Compared to this previous research, the BB approach has the advantage of being fully experimental, does not rely on any a priori knowledge about the sample composition and the experiment conditions and deals with scattering coming from the sample as well as from the experimental apparatus.

A different approach is described in [30], where very compact polycapillary neutron collimators are positioned between the sample and the detector, thus absorbing the scattered neutrons before they can hit detector. This however requires longer exposure times due to the large number of neutrons rejected by the collimator.

This paper concerns an expansion of our previous work [25], in which the proof of concept of the experimental method based on the BB correction was presented. The aim of this paper is twofold. Firstly, we fully describe the method with focus on the algorithm and data treatment and secondly, we provide a quantitative validation on both experimental measurements and simulations. The experimental setup with specific focus on neutron tomography, the mathematical formulation of the correction and the relevant image processing are detailed. We show the performance of the proposed approach in attenuating scattering and systematic bias related artifacts in 5 different CT datasets of hydrogenous and non-hydrogenous scattering materials acquired at the NEUTRA [31] and ICON [32] beamlines at the Paul Scherrer Institut (PSI).

## 2. Principle

In the following of this section, we present the developed methods for scattering and bias correction in neutron tomography using BB grids. We refer to the scattering component due to the collided neutrons with the sample as “sample scattering”, while the component due to the systematic biases, comprising neutron scattering with the experimental apparatus and the back reflected light due to the camera box configuration, will be referred as “background scattering”. Throughout the paper, we will refer to the proposed method as “BB correction”. First, we describe the acquisition protocol that includes the additional measurements required to estimate the scattering components. Then, the mathematical formulation of the normalization to reference images is rewritten, including the estimated components into the image models. Finally, we describe the image processing operations necessary to estimate the scattering component from the experimental measurements.

### 2.1 Nomenclature

Meaning of subscripts and superscripts

*I*_*n*_ Sample image for the n^th^ sample position

*I*_*OB*_ Open beam image

*I*_*DC*_ Dark current image

*I*_*n*,*BB*_ Sample image with BB pattern for the n^th^ sample position

*I*_*OB*,*BB*_ Open beam image with BB pattern

$I_{n}^{S}$ Sample scattering

$I_{BG}^{S}$ Background scattering

$I_{n,BB}^{S}$ Sample scattering computed from images with BBs

$I_{BG,BB}^{S}$ Background scattering computed from images with BBs

General symbols

I* Measured image

$\tilde{I}$ Normalized image

Other symbols

D Dose operator (a scalar value representing the intensity of the incoming beam. Often the average pixel intensity of a region outside the sample on all projections)

*τ*_*BB*_ ∈ [0,1] transmission parameter, describing mean transmission pattern of an image with a BB pattern (fraction value)

### 2.2 BB image acquisition

In neutron computed tomography, projection images of the sample are acquired from different views using a neutron source. The sample is turned with small angular increments around the acquisition axis until at least 180° or 360° have been covered. The traditional acquisition protocol also requires references images to be used for image normalization: the open beam images ($I_{OB}^{*}$) where the image is obtained without any sample in the beam and the dark current images (I_DC_) where the images are obtained in the absence of beam illumination, measuring background noise and camera offset.

For the measurements of the sample and background scattering components, we propose two additional sets of images to be acquired using a reference frame with and without the sample in the beam, immediately before tomography. In both cases, images are acquired with an interposed frame containing neutron absorbers, black bodies. The BBs are opaque to neutrons; therefore, the measured neutron flux behind them can be interpreted as the scattered neutrons component. Size and position of the BBs depend on the size of the field of view (FOV). For a detector setup with a FOV of 150 mm x 150 mm we used cylindrical BB objects made of ^10^B_4_C (2.5 mm diameter and 3 mm length) mounted on a rectilinear grid using an aluminium frame with their longitudinal axis oriented as the neutron beam. The BBs are arranged on a uniform rectangular grid cut by water jet and is 10 mm thick, holding the BBs with a centre-to-centre spacing of 25 mm. The frame design is realized with the aim of minimizing the amount of material that could introduce as well some scattering effect, potentially affecting the measurements.

**Fig 1:**
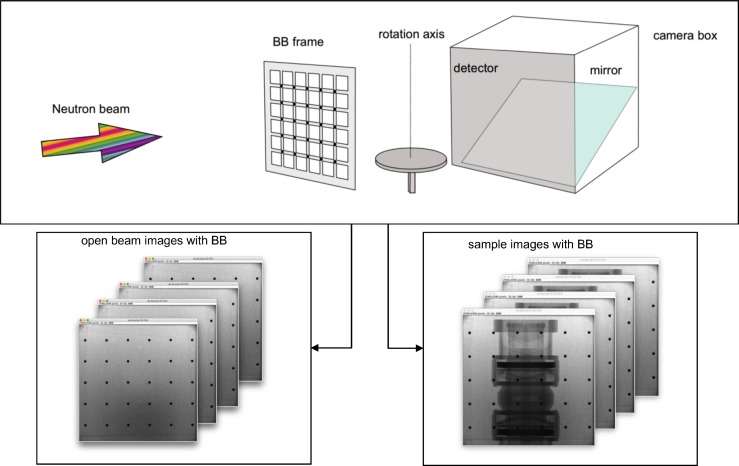
Schematic of image acquisition with interposed BB frame. Two set of images are acquired without the sample (left) and with the sample in the beam (right).

In the experimental setup (Fig 1) the BB frame is first placed in a close position to the sample holder. Then, a set of open beam images with interposed the BB grid are acquired. This measurement aims at recording the scattering component of the background only, i.e. neutrons scattered at the shielding or the instrumentation and the light reflected from the mirror back to the scintillator.

Then, the sample is placed onto the holder and a set of images with interposed BBs grid is acquired. In the optimal setup, the sample is centred in the neutron beam and its longitudinal axis aligned to the column detector, and the BB grid is placed so that one BB line in the radiograph is superimposed to the sample longitudinal axis.

Asymmetric samples cause different sample scattering components depending on the viewing angle. This must be considered when reference images are acquired with the sample. Thus, the BB reference acquisition with samples becomes a sparse CT scan. The reference scan can be performed using much fewer projections than the planned CT scan as the sample scattering is a very smooth function that varies much less than the sample projections. The sparse reference scan aims at saving experiment time with ideally little or no loss of quality.

The BB frame is finally removed to perform the intended tomography scan of the sample.

### 2.3 Image normalization using BB correction

The conventional method to normalize the acquired images using references is often called open beam correction and requires:

- the dark current image (*I*_*DC*_),
- the measured sample image ($I_{n}^{*}$) and the tomographic n^th^ position. Under the assumption of pure transmission, the measured sample image can be modeled as the sum of the sample image and the dark current: $I_{n}^{*}=I_{n}+I_{DC}$
- the measured open beam image ($I_{OB}^{*}$), similarly modeled as $I_{OB}^{*}=I_{OB}+I_{DC}$

Sample images (*I*_*n*_) are then normalized according to:

$$\frac{{\tilde{I}}_{n}}{{\tilde{I}}_{OB}}=\frac{I_{n}}{I_{OB}}∙\frac{D(I_{OB})}{D(I_{n})}=\frac{\left(I_{n}^{*}-I_{DC}\right)}{\left(I_{OB}^{*}-I_{DC}\right)}∙\frac{D(I_{OB}^{*}-I_{DC})}{D(I_{n}^{*}-I_{DC})}$$ (1)

Where the dose operator D computes an average beam intensity on the same open beam region of interest (ROI) in $I_{n}^{*}$ and $I_{OB}^{*}$, thus compensating in Eq 1 for dose fluctuation in the neutron beam intensity. In common practice, a stack of open beam images and a stack of dark current images are acquired and then averaged with the aim to increase statistics and decrease noise.

To take into account the scattering contribution from the interaction of the beam with the sample and from the imaging setup (camera box, walls and surrounding structural materials) we reformulate the image normalization formula Eq 1 as follows.

Let the measured sample n^th^-image denoted as $I_{n}^{*}$ be modeled as the sum of the “true” sample image *I*_*n*_, a scattering component $I_{n}^{S}$ and the dark current signal *I*_*DC*_ with contributions from the sample and background scattering:

$$I_{n}^{*}=I_{n}+I_{n}^{S}+I_{DC}$$ (2)

Similarly, let the measured open beam image $I_{OB}^{*}$ be modelled as the sum of the open beam image *I*_*OB*_, the background scattering to the camera system $I_{BG}^{S}$ and the dark current signal *I*_*DC*_:

$$I_{OB}^{*}=I_{OB}+I_{BG}^{S}+I_{DC}$$ (3)

The image referencing formula in Eq 1 can be rewritten taking into account these scattering components as:

$$\frac{{\tilde{I}}_{n}}{{\tilde{I}}_{OB}}=\frac{I_{n}}{I_{OB}}∙\frac{D(I_{OB})}{D(I_{n})}=\frac{I_{n}^{*}-I_{DC}-I_{n}^{S}}{I_{OB}^{*}-I_{DC}-I_{BG}^{S}}∙\frac{D(I_{OB}^{*}-I_{DC}-I_{BG}^{S})}{D(I_{n}^{*}-I_{DC}-I_{n}^{S})}$$ (4)

In the BB correction, we estimate the scattering component in Eq 4 from the experimental BB data and compensate for the dose variation. We rewrite the mathematical formulation referring to the experimental BB data as follows.

Let $I_{OB,BB}^{*}$ and $I_{n,BB}^{*}$ be the measured images with BB with and without the sample, respectively. These images are processed as described in the following section to estimate the scattered neutrons components $I_{BG,BB}^{S}$ and $I_{n,BB}^{S}$, respectively.

Let the mean transmission pattern for images acquired with BB frame be denoted as *τ*_*BB*_ ∈ [0,1]. This parameter takes into account that the images acquired with BBs have a lower mean transmission compared to the images acquired without the frame, due to the presence of the BBs and the holding frame.

We can then write the relationship between the scattering components measured with BB patterns and the scattering component of Eq 4 as:

$$\begin{array}{c} {\tilde{I}}_{n,BB}^{S}=\tau_{BB}I_{n}^{S}\\ {\tilde{I}}_{BG,BB}^{S}={\tau_{BB}I}_{BG}^{S} \end{array}$$ (5)

where the ~ symbol indicates that images are normalized through the dose operator.

We reformulate Eq 5 including explicit normalization as:

$$I_{n}^{S}=\frac{{\tilde{I}}_{n,BB}^{S}}{\tau_{BB}}=I_{n,BB}^{S}\frac{D(I_{n}^{*}-I_{DC})}{D(I_{n,BB}^{*}-I_{DC}-(1-\frac{1}{\tau_{BB}})I_{n,BB}^{S})\tau_{BB}}$$ (6)

$$I_{BG}^{S}=\frac{{\tilde{I}}_{BG,BB}^{S}}{\tau_{BB}}=I_{BG,BB}^{S}\frac{D(I_{OB}^{*}-I_{DC})}{D(I_{OB,BB}^{*}-I_{DC}-(1-\frac{1}{\tau_{BB}})I_{BG,BB}^{S})\tau_{BB}}$$

where the $\left(1-\frac{1}{\tau_{BB}}\right)I_{x,BB}^{S}$ term, with *x* = *OB* or n, compensates for the decrease in transmission due to the presence of the BB frame.

The image referencing formula Eq 4 can be finally explicitly rewritten including the scattering components in Eq 6 as follows:

$$\frac{{\tilde{I}}_{n}}{{\tilde{I}}_{OB}}=\frac{I_{n}}{I_{OB}}∙\frac{D(I_{OB})}{D(I_{n})}=\frac{I_{n}^{*}-I_{DC}-I_{n,BB}^{S}\frac{D(I_{n}^{*}-I_{DC})}{D(I_{n,BB}^{*}-I_{DC}-(1-\frac{1}{\tau_{BB}})I_{n,BB}^{S})\tau_{BB}}}{I_{OB}^{*}-I_{DC}-I_{BG,BB}^{S}\frac{D(I_{OB}^{*}-I_{DC})}{D(I_{OB,BB}^{*}-I_{DC}-(1-\frac{1}{\tau_{BB}})I_{BG,BB}^{S})\tau_{BB}}}∙\frac{D(I_{OB}^{*}-I_{DC}-I_{BG,BB}^{S}\frac{D(I_{OB}^{*}-I_{DC})}{D(I_{OB,BB}^{*}-I_{DC}-(1-\frac{1}{\tau_{BB}})I_{BG,BB}^{S})\tau_{BB}})}{D(I_{n}^{*}-I_{DC}-I_{n,BB}^{S}\frac{D(I_{n}^{*}-I_{DC})}{D(I_{n,BB}^{*}-I_{DC}-(1-\frac{1}{\tau_{BB}})I_{n,BB}^{S})\tau_{BB}})}$$

## 3. Data processing

### 3.1 Analysing BB reference images

In this section, we describe the processing workflow to derive the scattering components $I_{n,BB}^{S}$ and $I_{BG,BB}^{S}$ from the measured images $I_{n,BB}^{*}$ and $I_{OB,BB}^{*}$. The assumption is that those components are smooth functions that can be described by parameters estimated using the image intensity from the points covered by the BBs in the measured images with the BB frame installed.

The workflow is divided into two steps; mask generation and interpolation. The first step requires image segmentation to detect the BB positions. Starting from the averaged measure of the stack of $I_{OB,BB}^{*}$ (image obtained with the BB frame and no sample) the processing pipeline is the following:

1. Computation of a normalized image with BB: (IOB,BB*−IDC)(IOB*−IDC) to compensate for the beam shape.
2. Otsu-thresholding [33] to the image obtained in 1, resulting in a binary image with white values for all pixels above the threshold. All objects are labelled and morphological cleaning is applied by removing the objects with area less than 20 pixels, considered due to the presence of noise. This processing produces a binary mask highlighting all BBs.
3. Labelling of single BBs in the original image ($I_{OB,BB}^{*}$) and computation of the weighted centre of mass for each item.
4. Solid discs with defined radius (~ half the dimension of the BB) are drawn at each BB centre. This produces a binary image to be used as a mask for the following interpolation.

The mask is used to obtain the pixel values and their coordinates in the $\left(I_{OB,BB}^{*}-I_{DC}\right)$ and $\left(I_{n,BB}^{*}-I_{DC}\right)$ images. Frome these, one has to estimate the parameters of the interpolation function to compute images representing the scattering components: $I_{BG,BB}^{S}$ and $I_{n,BB}^{S}$, respectively.

Two interpolation functions are investigated: (i) 2D polynomials with up to second order terms and (ii) thin plate splines (TPS).

In the case of 2D polynomial interpolation, the pixel values corresponding to the points under the mask are first filtered to remove possible outliers using basic sigma clipping with ±2σ. The interpolation formula to compute the scattering images can be written as:

$$I(x,y)=a+bx+cx^{2}+dxy+ey+fy^{2},$$

where x and y are the image coordinates of the points used for interpolation and *I(x*,*y)* are the corresponding grey values. By collecting the *N* points (pixel coordinates and intensity) under the BB mask, one creates a *N×6* matrix, to be solved to obtain the *{a*,*b*,*c*,*d*,*e*,*f}* parameters. The typical number of N is around 2000 points, for a setup with up to 150mm x 150mm, in which 25 BBs are segmented and a sub-region with circular shape with 5 pixels radius is chosen for each BB.

The QR algorithm is applied to solve the system of linear equations. The relative root mean square error between the image values *I(x*_*n*_,*y*_*n*_*)* under all the selected *N* points and their interpolated values I_int_(x_n_,y_n_) was then computed: $err=\sqrt{\frac{1}{N}\sum_{n=1}^{N}{\left(\frac{I_{int}(x_{n},y_{n})-I(x_{n},y_{n})}{I(x_{n},y_{n})}\right)}^{2}}$

For TPS interpolation, the median values of the pixels under each detected BB are computed and used for interpolation. In this case, the solution of the interpolation is exact in the interpolation points and smooth in the intermediate positions. According to [34], the interpolant is of the form: $I_{int}(x,y)=\sum_{k=1}^{K}A_{k}d_{k}^{2}logd_{k}+a+bx+cy$, where K are the number of detected BBs with *(x*_*k*_, *y*_*k*_*)* weighted centre and $d_{k}^{2}=(x-x_{k})+(y-y_{k})$. The TPS coefficients *A*_*k*_, *a*, *b* and *c* are obtained by solving the linear system:

$$\sum_{k=1}^{K}A_{k}d_{k}^{2}logd_{k}+a+bx+cy{|}_{\left(x,y)=(x_{k},y_{k}\right)}=I(x_{k},y_{k})\sum_{k=1}^{K}A_{k}=0$$

$$\begin{array}{c} \sum_{k=1}^{K}A_{k}x_{k}=0\\ \sum_{k=1}^{K}A_{k}y_{k}=0 \end{array}$$

This system yields to a distance matrix, symmetric but not positive definite (i.e. a special case of a Hollow matrix). The SVD algorithm is applied to solve the linear system.

Either way, the scattering components $I_{BG,BB}^{S}$ and $I_{n,BB}^{S}$ are obtained, for the angular position at which projection images with interposed BB frame were taken. The BB projections $I_{n,BB}^{*}$ are acquired much sparser than the sample projections. Therefore, we used pixel-wise linear interpolation between the two adjacent BB projections to compute the sample scattering at each tomographic angular position.

### 3.2 BB correction in the CT reconstruction pipeline

The scattering correction with the described method is implemented in our in-house software MuhRec [35]. This is an open source multi-platform tool developed at PSI for neutron and X-ray CT reconstruction based on filtered back projection [36] with a modular architecture that allows custom configuration of the processing pipeline. A dedicated module taking care of the relevant processing and the normalization formula (*BBLogNorm*) is implemented and can be included in the pre-processing chain. A configuration dialog helps users to select BB reference images and to tune parameters. The *BBLogNorm* module is made available in the latest MuhRec release [37].

The module is written in C++ and parallel computing is implemented using OpenMP [38]. The tnt library [39] is used to solve the linear equation systems.

## 4. Verification data

### 4.1 Experiments

Complete CT data sets including reference images were acquired of five different test samples at the NEUTRA and ICON neutron imaging beamlines at PSI. The samples and the acquisition conditions are summarized in Table 1.

**Table 1:** Sample descriptions.

#	Sample description	Shape	Size [mm]	BB images	Beamline	Rotation axis to scintillator distance [mm]	Exposure time [s]	Pixel spacing [μm]	Expected attenuation coefficient [cm^-1^]
1	Water column in Al container	cylinder	10 mm (external diameter), 8 mm (internal diameter)	25 images, evenly distributed over 360°	NEUTRA	23	23	46	3.6
2	Copper sample #1	cube	25x25x25 mm	25 images, evenly distributed over 360°	NEUTRA	23	23	46	0.94
3	Copper sample #2	Cylinder with holes	25 mm diameter of the sample, 6 mm diameter of the holes	25 images, evenly distributed over 360°	NEUTRA	23	23	46	0.94
4	Lead alloy sample (96%Pb and 4%Sb)	block	50 x15 x 100 mm	625 images over 360°	NEUTRA	32	8	67	0.335±0.01
5	BNC connector	Cylinder	14.5 mm diameter	375 images over 360°	ICON	20	80	13.5	various

Hydrogenous and non-hydrogenous materials were chosen for their well-known scattering properties: water (sample 1), copper (samples 2,3), lead alloy (sample 4) and a BNC connector, a sample assembled with metallic and plastic components (sample 5).

For samples 1 to 3, their averaged attenuation coefficients were estimated considering the thermal spectrum at NEUTRA (mean wavelength 1.8Å) and the ^6^LiF/ZnS scintillator efficiency. The two copper samples were packed together one of top of the other with Al foil and imaged in the same round to save experiment time. In all cases, 626 tomographic steps were acquired evenly sampling the 0-360° range. Sparse tomography with interposed BB frames was acquired by sampling the 0-360° range with 26 uniformly distributed projections.

Sample 4 and 5 were then acquired to test the effect of the interpolation of the scattering component for missing angles in sample with different shape and attenuation properties. Projections with the sample and interposed BB frame were in these cases acquired for more projections angles. For sample 4, the expected attenuation coefficients were computed experimentally by measuring the attenuation of images obtained with a pencil neutron beam and with the sample positioned 5 meters away from the detector. Please refer to [25] for further details.

For the measurements taken at NEUTRA, 100 μm thick ^6^LiF/ZnS with Andor Neo sCMOS, 2160 x 2560 pixels camera were used at measuring position II. For the measurements taken at ICON, the micro-setup was adopted (27x27mm^2^ FOV), featuring 20μm thick GadOx scintillator, Andor DW436K CCD camera with 2048x2048 pixels resulting in effective pixel size of 13.5μm.

### 4.2 Simulated datasets

Simulated CTs were computed to furthermore verify the validity of the proposed correction method and to quantify the ability of the interpolation schemes to describe the shape of the sample scattering. An *ad hoc* Monte Carlo model based on McStas [40] was developed, as described in [41], including the NEUTRA Maxwellian spectrum, the beamline geometry and the position-sensitive detector (PSD) component. The simulated sample was a powder-like block of Copper (2.5x2.5x5mm), non-perfectly aligned to the detector (3° tilt along beam direction and 0.2° tilt along the detector horizontal direction) [42]. An additional component simulating the BB grid was implemented by designing a 6x6 regular grid of beam stoppers with size 2.4x2.4mm, interposed in the simulation between the sample and the beam.

With this setup, three tomographic datasets were simulated sampling the 0-360° range with 626 equidistant angular positions (pixel spacing 15.6 μm): (i) one simulated tomography without BBs, thus obtaining projections with contributions from both scattering and transmission, (ii) one simulated tomography without BBs but ignoring the scattered neutrons hitting the detector, thus obtaining a “ground truth” measurement including only transmission, and (iii) one tomography with BBs, used to estimate the scattering components through the described approach. Of note, in the McStas simulations there is no background scattering effect, thus the ability of the method to compensate for the sample scattering only will be evaluated.

Raw data from the simulation were converted into image files and imported in MuhRec for further processing.

### 4.3 Data evaluation

Raw data were reconstructed using MuhRec. For all experimental datasets, the basic processing pipeline included: (i) normalization to reference images, (ii) spot cleaning, (iii) ring cleaning, (iv) projection filter and (v) back projection for parallel beam geometry.

Beam hardening was not negligible for the water sample, and the formula necessary to correct polychromatic to monochromatic attenuation was estimated as a third order polynomial: *y* = 0.99*x* + 0.03*x*^2^ + 0.0009*x*^3^. This step was applied before the projection filter in the reconstruction pipeline.

All samples were reconstructed with and without the described scattering correction, with the polynomial and TPS interpolation schemes. The percentage decreases in transmission were estimated of 0.7% due the presence of BBs and of 2.0% due to the presence of the holding frame, thus the *τ*_*BB*_ parameter was set to 0.97 [25].

For the water sample, the raw data were furthermore processed with the approach described [27], to compare the performance of the proposed method with previous research. The [27] method was chosen as its application was reported to successfully compensate for scattering related artifacts in samples containing water as main scatterer [43] [44] [45] [46]. Pre-defined NEUTRA spectrum, detector information and the material information (cross sections and simulated point scattered function, PScF) were loaded. The background scattering was empirically set a constant value (1200, corresponding to around 2.5% of the open beam). Corrected, referenced and normalized projections were imported to MuhRec and reconstructed. The obtained results were finally compared with those obtained with the proposed approach.

In order to investigate the impact of reducing the number of reference images, we reconstructed the lead sample several times with a decreasing number of BB projections each time. This analysis was performed because the number of BB projections should be minimized to save experiment time. In particular, 9 different configurations of BB projections were considered:

1. 625 BB projections, resulting into the reference dataset for scattering estimation
2. 25 BB projections, distributed into the 360° tomographic range with the first projection at 0° and 14.4° distance between consecutive projections
3. 13 BB projections, distributed into the 360° tomographic range with the first projection at 0° and 28.8° distance between consecutive projections
4. 7 BB projections, distributed into the 360° tomographic range with the first projection at 0° and 57.6° distance between consecutive projections
5. 4 BB projections, distributed into the 360° tomographic range with the first projection at 0° and 115.2° distance between consecutive projections
6. 4 BB projections, distributed into the 360° tomographic range with the first projection at 14.4° and 86.4° distance between consecutive projections
7. 2 BB projections, distributed into the 360° tomographic range with the first projection at 0° and 230.4° distance
8. 1 BB projection at angle 0°
9. 1 BB projection at angle 270°

The first case represents the ground truth and the reference for the other cases, as from a complete scan of 625 images with BBs it is possible to estimate the scattering component for each tomographic position. In cases 2 to 7, pixel-wise linear interpolation is applied to obtain the scattering component of all tomographic position. In cases 8 and 9, the same correction estimated from the single image with BB is computed and applied to all tomographic positions.

Analogously, a simplified analysis was performed on the BNC connector (sample #5). Our hypothesis here is that when objects with simpler geometry are scanned, cylindrical in this case, then the scattering shape varies only little, possibly negligibly, when varying the projection angle. Thus, the scheme for the sparse tomography with BBs could be furthermore reduced to a minimal amount of required measurements and accordingly experiment time. The following configurations of BB projections were considered:

1. 375 BB projections, distributed into the 360° tomographic range with 0.96° steps
2. 15 BB projection, distributed into the 360° tomographic range with 24° steps
3. 1 BB projection at angle 0

As regards to the simulated tomographies, the sample scattering contribution was calculated by subtracting the projections obtained with transmission and scattering and those obtained only from transmitted neutrons. The sample scattering was then estimated according to the described technique applying the polynomial and the TPS interpolation and compared to the nominal value. CT reconstruction was finally performed.

The statistical significance of the results presented in the following section is intended when the p value results <0.01, for the rank sum test.

## 5. Results and discussion on experimental data

### 5.1 Water sample

#### 5.1.1 CT reconstruction

**Fig 2:**
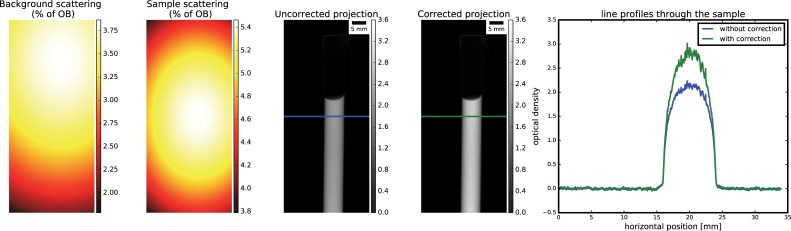
Computed background and correction on radiography for the water sample.

**Fig 3:**
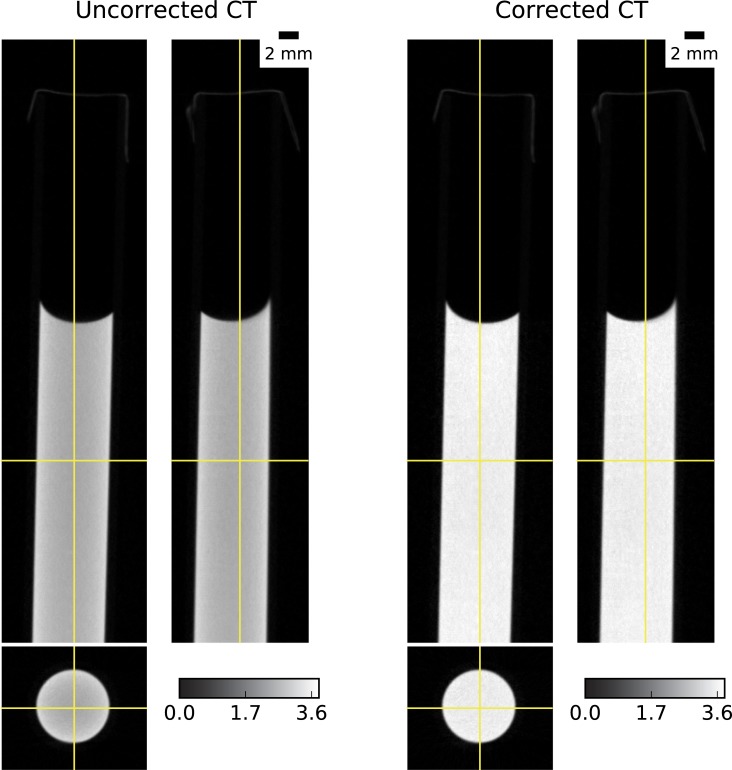
Uncorrected and corrected CT reconstruction of the water sample.

**Fig 4:**
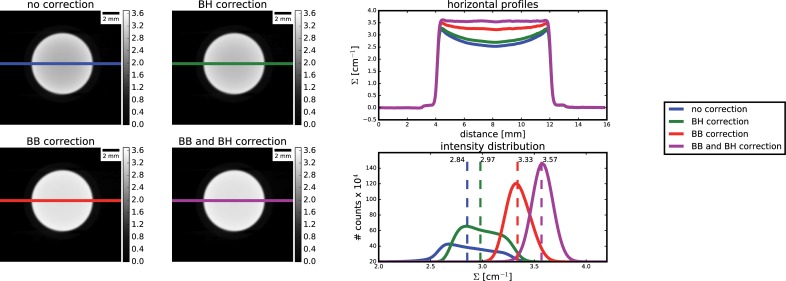
Results on the water sample: combined effect of BB correction and BH correction on the computed attenuation coefficients.

In Figs 2, 3 and 4, we show the results obtained for the water sample. The computed background and sample scattering using the 2^nd^ order polynomial scheme ranged from around 1.8 to 3.8% of the open beam and from around 3.8 to 5.5% of the open beam, respectively (Fig 2 left panels). The effect of the scattering compensation resulted in higher density on corrected projections, compared to the uncorrected (Fig 2, right panels).

On CT reconstruction, qualitatively comparison shows that corrected CT have higher attenuation coefficient, closer to the expected 3.6 cm^-1^, and the cupping artefacts seem to be attenuated (Fig 3). More quantitative analysis on CT reconstruction is presented in Fig 4, where we also present the combined effect of scattering and beam hardening correction. By applying the scattering correction alone, mean attenuation coefficients rise from around 2.84 to 3.34 cm^-1^, together with a noticeable mitigation of the cupping artefact. When correcting only for beam hardening, a slight improvement on attenuation coefficient is registered (mean value from 2.84 to 2.97 cm^-1^), while almost negligible effect is seen on the cupping artefact. When finally correcting for both effects, we obtain a narrower distribution of attenuation coefficients, with mean value around the expected attenuation (3.57 cm^-1^), and the cupping effect is more efficiently compensated. In this analysis, polynomial interpolation scheme was adopted to approximate the scattering component.

#### 5.1.2 Evaluation of the interpolation scheme

**Fig 5:**
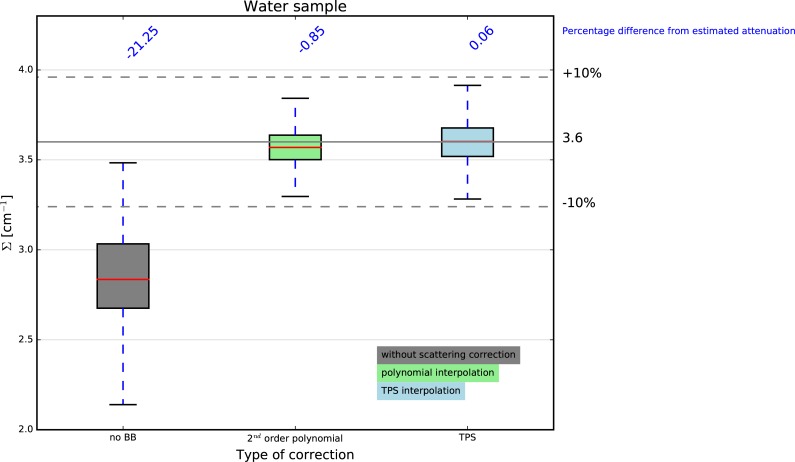
Effect of different interpolation scheme on the attenuation coefficients for the water sample.

As regards the interpolation scheme, the overall performance of the correction does not seem to be influenced by the choice of one or the other, polynomial or TPS (Fig 5).

The median values of the intensity move towards the expected values when applying the scattering correction with small although significant differences when using different interpolation schemes. The attenuation coefficients for water are underestimated by 21.52% for the uncorrected case, while the absolute error is less than 1% when the proposed correction is applied. A slightly better result is obtained when adopting the TPS (0.13%) compared to the polynomial (-0.79%) interpolation scheme.

#### 5.1.3 Comparison with previous research

**Fig 6:**
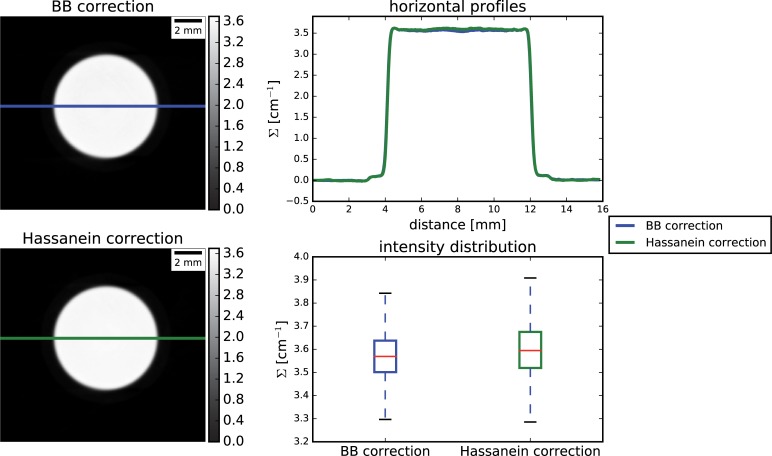
Comparison between the BB correction and the method proposed by Hassanein [27] for the water sample.

The results on the water sample (sample 1) obtained with the proposed approach are compared with those obtained with the method described by Hassanein [27] in Fig 6.

As shown, both are able to compensate for cupping related artefacts and provide attenuation coefficients close to the expected. The horizontal line profiles of the averaged slice are almost indistinguishable. The intensity distribution of the grey values within the entire sample resulted statistically significantly different however very similar medians values, 3.59 and 3.57 cm^-1^, and with only a slightly broader distribution for the [27] method compared to the BB correction (interquartile ranges 3.52 to 3.68 and 3.5 to 3.64, respectively). The main advantage of the proposed approach is that, being fully experimental, no a priori knowledge of the sample or the experimental condition is required, while for [27], significant a-priori knowledge is necessary: beamline spectrum, material composition and shape, detector characteristics, Monte-Carlo simulations of the PScF. Being unrelated to the knowledge of the sample shape and composition, the proposed correction can be then easily applied potentially to any sample undergoing neutron imaging. On the other hand, the BB correction intrinsically requires an increase of the experimental time, even if moderate, as an additional sparse tomography with BBs has to be acquired.

### 5.2 Copper samples

#### 5.2.1 CT reconstruction

**Fig 7:**
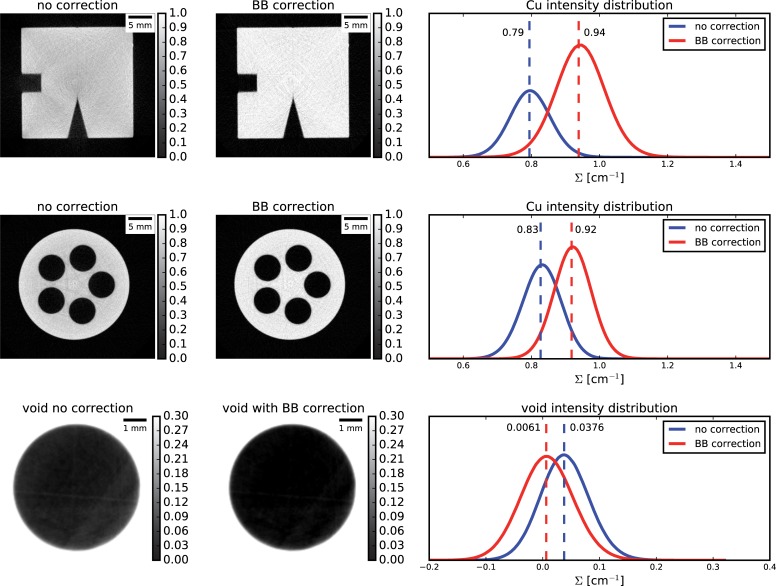
Results for the copper samples.

Results on CT reconstruction for the copper samples are shown in Fig 7. For the first sample (first row), higher attenuation coefficient and more homogeneous intensity distribution within the sample were found, however together with a broader intensity distribution, suggesting that the proposed correction might slightly amplify the noise as side effect.

For the cylindrical Cu sample (middle and bottom rows of Fig 7), the algorithm again proved to efficiently correct for scattering, resulting in higher attenuation coefficient, closer to the expected value (~0.94cm^-1^). The effects of the correction are furthermore noticeable in the voids within the sample, where values closer to zero are computed thanks to the sample and background scattering removal.

#### 5.2.2 Evaluation of the interpolation scheme

**Fig 8:**
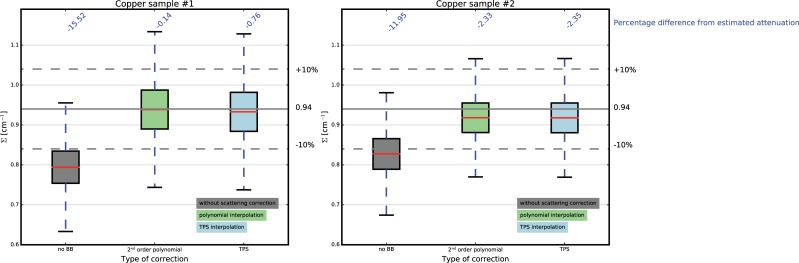
Effect of the interpolation scheme on the computed attenuation coefficients for the copper samples.

Fig 8 shows the results for the two copper samples in terms of intensity distribution of the attenuation coefficients, obtained with and without BB correction and by applying the two interpolation schemes.

The underestimation of the attenuation coefficients when scattering is not compensated is about 15.53% and 11.95% for the two samples, respectively. When applying corrections, for the first sample the underestimation is below 1%, while for the second is around 2.35%, with slightly better results when using the polynomial compared to the TPS interpolation approach.

### 5.3 Lead sample

**Fig 9:**
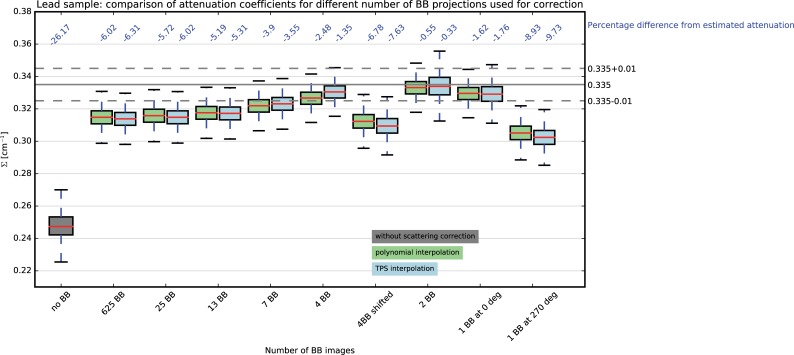
Attenuation coefficients distribution obtained with BB correction for the lead sample, by varying the number of BB images.

On the lead sample (sample #4), we investigate the effect of the correction when varying the number of projections acquired with BBs and by applying the two interpolation approaches. In [25], the same sample was evaluated and scattering related cupping artefacts were proven to be successfully compensated, leading to homogeneous results in the reconstructed data. Fig 9 shows the distribution of the attenuation coefficients that are obtained without correction and with correction obtained by varying the number of projections acquired with the interposed BB frame. Without correction, the attenuation coefficients are underestimated of 26.17% compared to the expected value. In general, the results obtained with the two investigated interpolation schemes vary only slightly, although statistically significantly (rank sum test). This confirms the results obtained in the water and copper samples (samples #1,2,3), suggesting that both interpolation schemes successfully describe the shape of the scattered neutrons.

**Fig 10:**
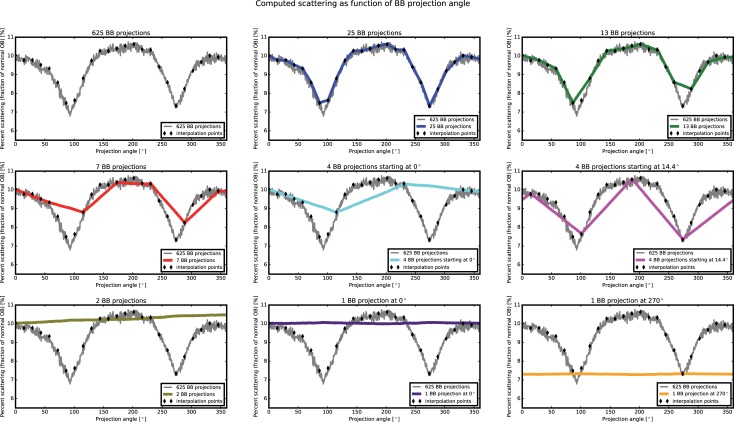
Effect of the interpolation of missing angles on the computed scattering components.

In the case of a complete scan of 625 images with BBs, the underestimation is significantly reduced to 6.02% and 6.31%, for polynomial and TPS interpolation, respectively. When using a sparse tomography of 25 projections with BBs uniformly, a very similar result is found, with a percentage error of 5.72% and 6.02% for polynomial and TPS interpolations, respectively. By diminishing the number of BB images till the case 7 projections, an effect of progressively reduced underestimation can be noticed. When using 4 BB projections, the results on the attenuation coefficients varies significantly between the two configurations, with lower underestimation of the attenuation coefficients for the first case, where the BB scan started at 0°, compared to the second case, where the BB scan started at 14.4°. The minimal underestimation below 1% resulted when using only 2 BB images. When using only one BB image at the tomographic position 0°, an underestimation below 2% is computed, while when using only one BB image at 270°, the underestimation is around 10%. To understand this behaviour, we show in Fig 10, the estimated background as function of the BB projection angle, for the different BB acquisition schemes (using polynomial interpolation).

For each projection angle, the median value of a region with size 20x30 pixels around one BB position in the central area of the sample, normalized by the median value of the open beam and dose corrected, is computed and plotted for each angular position. In grey, the computed values for the 625 BB projections are plotted. In this case, the computed scattering lies in the range 2.8% to 10.8% of the OB values, with a “quasi-sinusoidal” shape that describes the relation between the neutron scattering and the projection angles.

By reducing the number of BB projections, fewer interpolation points are progressively considered. When using the 25 BB images, the shape of the scattering component is well preserved almost in every projection angle, except for an overestimation between the 7^th^ and 8^th^ BB images, corresponding to the tomographic angles 86.4° and 100.88°. With 13 BB projections, the overestimation of the background becomes more pronounced in the angle ranges 86.4° to 115.2° and 244.6° to 273.6°. Overestimation of the scattering component due to the interpolation scheme is even more pronounced in the case of 7 BB projections and 4 BB projections starting at the 0° tomographic position and maximum in the case of 2 BB images, as the correction is above 10% of the OB values for all tomographic position. Conversely, when using 4 BB projections starting at 14.4° the scattering components are underestimated in most part of the tomographic range, except in the tomographic range 72° -115° where it is instead overestimated. Finally, when using only one projection at the position 0°, the scattering is again overestimated, while when using the projection at position 270°, the scattering component is underestimated with values around the 8% of the OB for all tomographic position.

In the BB configurations resulting in underestimation of the estimated scattering, lower attenuation coefficients are computed from the reconstruction compared to the reference case of a full tomographic scan with 625 BB projections and consequently to the theoretical values. Vice versa in all cases where the scattering was overestimated, the reconstructed CT resulted in higher attenuation coefficients, as shown in Fig 9. This numbers are closer to the expected attenuation coefficient, however artificially. This configurations should be carefully considered and especially avoided when the overestimation becomes more evident, in order to avoid introducing further biases.

### 5.4 BNC connector

**Fig 11:**
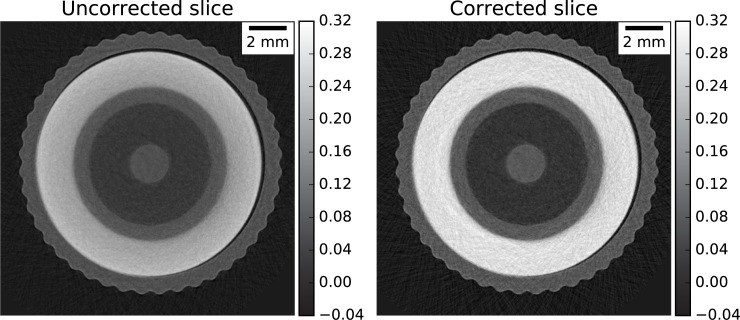
Exemplary slice for the reconstructed BNC sample without and with BB correction.

For the BNC connector (sample #5) an exemplary slice shows the effect on the scattering artefacts (Fig 11, right) and its correction using the entire dataset of BB images (Fig 11, left).

**Fig 12:**
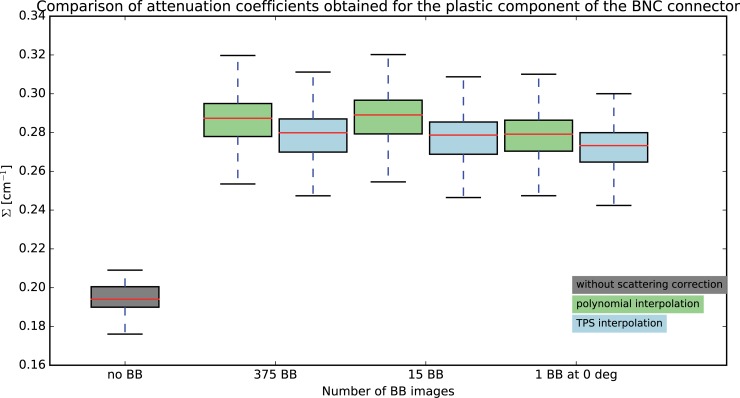
Attenuation coefficients distribution obtained the in plastic ring component of the BNC sample, obtained by varying the number of BB images.

As expected, the effect of the correction is noticeable at the plastic ring component, clearly showing the scattering related cupping effect in the uncorrected case. Further quantitative analysis is thus performed on a ROI included (10x10 pixels) in the plastic component. As shown in Fig 12, the attenuation coefficients of the plastic significantly increase from a median value of 0.194 cm^-1^ in the uncorrected case to around 0.28 cm^-1^ in the case of BB correction. The various BB configurations differ between each other only slightly, with maximum difference less than 3% between the 375 BB and 1 BB configurations, for both polynomial and TPS interpolation scheme. As expected, the difference is in attenuation coefficient when moving from 375 BB to 15 BB is negligible (<1%). Overall, the BB correction with polynomial interpolation resulted in slightly higher values compared to those obtained with the TPS interpolation.

## 6. Results and discussion on simulations

**Fig 13:**
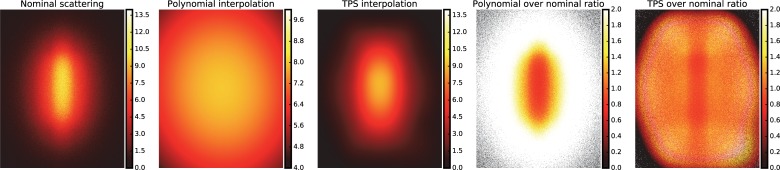
Scattering functions computed for the simulated datasets.

From the simulated tomography, the nominal sample scattering was computed as the pixel-wise image difference between the projections obtained with transmission and scattering and those obtained only with transmission. The nominal scattering ranged from 0 to 13.5% of the open beam, with the higher values in the sample region (Fig 13, first panel). The sample scattering estimated with the proposed method applying the 2^nd^ order polynomial and TPS are shown in the second and third panels of Fig 13, respectively. It can be noticed that the TPS better approximates the sample scattering shape and covers the same range as the nominal one (0–13.5%), compared to the polynomial approach (range 4–9.7%). However, for both interpolation schemes, the ratio of the interpolated background over the nominal scattering shows a distribution with values close to one over the sample area (Fig 13, fourth and fifth panel), suggesting that the approximation is mostly correct. In more peripheral regions, the TPS is able to follow the scattering shape, while the polynomial interpolation overestimates it.

**Fig 14:**
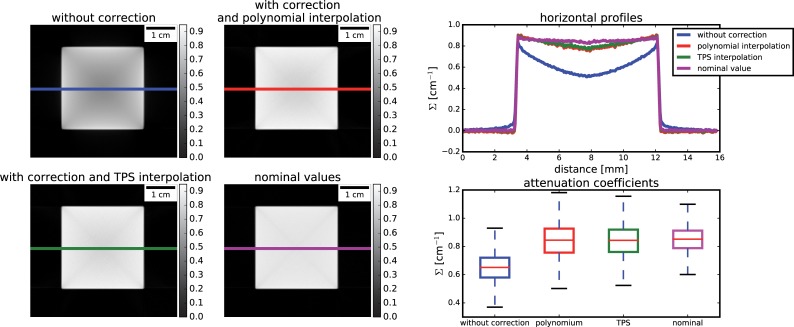
Results on CT reconstruction for the simulated datasets.

Fig 14 finally shows the relevant results of the reconstructed data. An averaged slice is shown together with a horizontal profile drawn at a middle position. A cupping effect together with underestimated attenuation coefficients is clearly shown on the reconstructed data without any correction. When applying the proposed correction, both interpolation schemes noticeably correct for the attenuation coefficients underestimation. The cupping effect is also mostly compensated, with residual errors due to the interpolation schemes of around 6% in the more central part of the sample compared to the nominal values.

With simulated tomographies, we were able to verify that (i) the scattering function is smooth, depends on the sample geometry and that it varies smoothly with the projection angles, (ii) both interpolation schemes are able to approximate this function. The TPS, being exact in the interpolant points, describe with higher fidelity the scattering shape, also outside the sample. However, in samples with adequate transmission (>10% of the open beam) the final effect on reconstructed data is independent to the interpolation scheme, (iii) in both cases, the effect of considering a relative small number points over the FOV for interpolation resulted in residual errors of around 6% in the middle position of the sample.

## 7. BB correction for dynamic processes

The BB correction can be applied as well in kinetic studies and it will be the object of future research. There are different approaches that can be chosen, depending on the specific measurement. In the general case, BB datasets should be acquired at the initial and the final condition, and at intermediate steps during the observation of the dynamic process if this can be accommodated. Under the assumption that the scattering function varies smoothly with time, the scattering function has to be interpolated in missing time steps. This interpolation is similar to the interpolation scheme for missing angular positions in case of tomography. Depending on the time resolution of the dynamic process and the expected variation of the scattering contribution, the acquisition of BB datasets can be either relaxed to the case when BB images are acquired only at the beginning and/or at the end of the dynamic process (for example at the dry and wet conditions in a wetting experiment), or stressed to the extreme case where all images are acquired with the interposed grid of BBs.

## 8. BB correction at finer resolution

The described design of the BB grid, holding 5x5 neutron absorber elements with 25 mm regular spacing, was chosen as a compromise between spatial resolution for scattering measurement and the portion of covered beam in a 150×150 mm^2^ FOV [25].

To correct for scattering with higher resolution, a valuable option is the “fine grid” scanning described in Boillat et al., obtained by shifting the BB grid in two directions perpendicular to the beam axis and acquiring several images with BB at the different position in the FOV. This way the background is measured with a finer spacing of 6.5mm instead of 25mm. This might in particular be useful for scattering contributions provided by sample features smaller than the 25 mm BB distance, as well as for samples with very low transmission, where the BB positions plays a decisive role for a successful correction.

We have also designed BB grids for the higher resolution setups available at PSI, e.g. the “micro set up” (27×27mm^2^ FOV) [17] and the “Neutron Microscope” (NM) [47]. In both cases, an Al plate is used as substrate and BBs are made of powder of ^157^Gd_2_O_3_. For the micro-setup, a regular grid is designed containing 6 by 6 BB with 0.5 mm diameter and 5 mm distance, this was the setting used for the measurements of the BNC connector (sample #4). For the NM, 11×11 BBs can be placed in a 5.5×5.5mm^2^ FOV, featuring 100μm diameter and 0.5mm BB separation. Thanks to these options, we can also apply the BB correction for experiments with higher resolution setup.

## 9. Conclusion

We have quantified and validated the BB correction method on several experimental and one simulated CT datasets. The data treatment has been detailed: image acquisition with the interposed BB frame, image processing to estimate scattering and biases function from the BB image and finally the analysis of the results, in order to provide with a robust and repeatable approach.

Our results show that correction of scattering from sample and background, and systematic biases is necessary to allow quantitative analysis of neutron imaging experiment results. We have investigated different hydrogenous and non-hydrogenous materials: water, copper, lead alloy and plastic. In all cases, the method was able to compensate for the cupping artefacts and as well to compute linear attenuation coefficient very close to those expected. With the simulation study, we were able to confirm that both choices of spatial interpolation investigated are valid to approximate the scattering function.

The BB correction offers a practical solution and has the advantage of not requiring any a priori knowledge of the experiment, neither sample composition nor experiment setup. This does however come at the expense of a slightly longer time to be allocated for the acquisition of the datasets with BBs. In order to minimize this additional experimental time, we have evaluated the effect of reducing the number of BB images to be used for correction. We can conclude that, in general, the choice of a sparse tomography for the BB datasets (one each 25 angular tomographic positions) provide a good sampling of the tomographic range (of typically 625 equidistant steps), without degrading the quality of the correction results. For samples with simple geometry (i.e. cylindrical), this scheme can be furthermore relaxed, until reaching the limit case of one only projection acquired with BBs with preserved efficacy.
